# 

**Published:** 1893-10-21

**Authors:** 


CONTENTS.

**Law, Medicine, and Humanity** - 33

**Hospital Saturday and Street Collections** - 34

**Empirical and Chemical Theories.** By Sir Benjamin Ward Richardson, M.D., F.R.S - 35

**The Medical Work of the Local Government Board.**—Vaccination - 36

**On the Cellular Structure of Living Organisms.** I. - 37

**The Hospital Clinic**—

St. Mary's Hospital.—On the Treatment of Aural Suppuration. II.—The Chronic Form - 41

Royal Infirmary, Edinburgh.—The Treatment of Erysipelas - 42

The Treatment of Ringworm at Bristol General Hospital and Elsewhere - 43

**Contemporary Theory and Research** - 38

**Annotations.**—Dr. A., Dr. B., and Dr. C.—The Howard Association—A Whisper to Women

**Notes and News** - 39

**Asylums in India** - 40

**The Institutional Workshop**— - 44

Hospital Administration.—Provincial, Scotch, and Irish Hospitals - 45

Hospital Construction.—New Home for Incurables, Newcastle-on-Tyne - 47

**Editor's Letter Box.—Scraps and Gleanings** - 48

**The Book World of Medicine and Science** - vii

**Medical Vacancies and Appointments** - ix

EXTRA SUPPLEMENT.—THE NURSING MIRROR.

**News from the Nursing World.**—Observant Visitors—Our Christmas Competitions—Things that are Wanted—Nurses for the Insane—Good Enough for the Poor—A Lasting Memorial—Lost Opportunities—India—A Pleasant Entertainment—Chicago and Ironwood—Short Items - xxi
**On the Nursing of Diseases of the Nervous System.**—III. Apoplexy and Aphasia, Cerebral Tumour and Abscess - xxiii
**Our Examinations** - xxiv
**The Nightingale Probationers** - xxiv
**In the District** - xxv
**Appointments** - xxv
**Nursing in South Africa.**—Robben Island, Cape Town. The Lunatic Asylum - xxvi
**Everybody's Opinion.**—Nursing Defects in Cottage Hospitals—A Trained Nurse in Difficulties - xxvii
**For Reading to the Sick.**—Bells - xxvii
**Minor Appointments** - xxvii
**Holiday Expeditions.**—I.— Mont Blanc - xxviii
**Wants and Workers** - xxviii
**The Book World for Women and Nurses** - xxix

